# Introduction to Morphological and Functional Evaluation of the Heart and Coronary Arteries

**DOI:** 10.5334/jbr-btr.1039

**Published:** 2016-02-12

**Authors:** Marco Francone, Gianluca De Rubeis, Francesco Cilia, Nicola Galea, Iacopo Carbone

**Affiliations:** 1 Sapienza University of Rome, IT Department of Radiological, Oncological and Pathological Sciences

## Introduction

In the last years, the number of clinical indications for the evaluation of the heart – with both computed tomography (CT) and magnetic resonance (MR) – exponentially grew. This evidence reflects the remarkable technological developments of both techniques allowing unprecedented spatial, temporal and contrast resolution levels and to comprehensively evaluate cardiac pathology, combining anatomical information with functional assessment and tissue characterization of myocardial diseases.

## Coronaries

In the field of non-invasive coronary artery imaging, CT has a prominent role, thanks to its superior spatial resolution (up to 0.3 mm with recently released scanners) and improved temporal resolution (up to 87 msec), and it is overall accepted better by the patient.

Theoretically, MR represents an ideal tool for coronary imaging, due to the lack of ionizing radiation or contrast agents, lack of beam-hardening artefacts related to the presence of calcifications and to its superb contrast-to-noise ratio and high temporal resolution (up to 50 msec) making coronary-related motion artefacts less likely to occur. As confirmed in a large number of metanalyses, however, this method is currently non-competitive with CT for the evaluation of coronary artery disease due to its overall high technical complexity with long scanning times (still consistently higher than five minutes) and due to the low spatial resolution (0-8-1 mm maximum), limiting reliable quantification of stenosis degree only to large-caliber proximal epicardial vessels.

As a matter of fact, cardiac MR is frequently required for the evaluation of origin and course of coronary arteries and to characterize benign vs malignant anomalies. In pediatric patients, Cardiac Magnetic Resonance (CMR) is also utilized in the follow-up of vasculitis (like Kawasaki disease) in which repeated follow-up scans are often required (with possible radiation overexposure) to monitor progression of giant coronary aneurisms.

## Cardiac and valvular function

Temporal resolution is the real gatekeeper for optimal cardiac functional and valvular imaging. Morphological assessment of valvular apparatus including evaluation of small vegetations or moving structures is also frequently required in combination with functional imaging and limited by spatial resolution constraints mostly for MR imaging.

As a consequence, cardiac MR is the current gold standard for both regional and global functional assessment including (bi-)ventricular volumes and ejection fraction and myocardial mass.

Although feasible and accurate, the role of CT in this application is limited by some important technical drawbacks, including the intrinsic increase of radiation dose related to the application of a retrospectively Electrocardiography (ECG)-gated scanning technique and to the relatively low frame-rate (i.e. temporal resolution) leading to the risk of over-estimating ventricular volumes (particularly End Systolic Volume ESV).

For the same reason, regional wall motion is also well assessed with MR, which also exploits the availability of a variety of techniques (from conventional cine-SSFP to real-time imaging and tagging sequences) allowing qualitative and semi-quantitative assessment of different components of intrinsic cardiac motion.

In valvular disease, combined morphological and functional assessment is also mandatory and both techniques are used in a variety of clinical setting in patients with valvular heart disease including pre-surgical evaluation (with TAVR) and assessment of prosthetic valves. CMR offers the added value of trans-valvular flow quantification which is particularly useful in right heart pathology and complex congenital heart diseases.

## Competing Interests

The authors declare that they have no competing interests.

